# The Th1/Th17 balance dictates the fibrosis response in murine radiation-induced lung disease

**DOI:** 10.1038/s41598-017-11656-5

**Published:** 2017-09-14

**Authors:** Alexandra Paun, Marie-Eve Bergeron, Christina K. Haston

**Affiliations:** 1 Meakins-Christie Laboratories and the Department of Human Genetics, McGill University Montreal, PQ Canada; 2 Meakins-Christie Laboratories and the Department of Medicine, McGill University, Montreal, PQ Canada; 3 Department of Medicine, McGill University, Montreal, PQ Canada; 4 Physics Department, I.K. Barber School of Arts and Sciences, The University of British Columbia Okanagan, Kelowna, BC Canada

## Abstract

Radiotherapy can result in lung diseases pneumonitis or fibrosis dependent on patient susceptibility. Herein we used inbred and genetically altered mice to investigate whether the tissue adaptive immune response to radiation injury influences the development of radiation-induced lung disease. Six inbred mouse strains were exposed to 18 Gy whole thorax irradiation and upon respiratory distress strains prone to pneumonitis with fibrosis presented an increased pulmonary frequency of Thelper (Th)17 cells which was not evident in strains prone solely to pneumonitis. The contribution of Th17 cells to fibrosis development was supported as the known enhanced fibrosis of toll-like receptor 2&4 deficient mice, compared to C57BL/6J mice, occurred with earlier onset neutrophilia, and with increased levels of pulmonary Th17, but not Th1, cells following irradiation. Irradiated Il17−/− mice lacked Th17 cells, and were spared both fibrosis and pneumonitis, as they survived to the end of the experiment with a significantly increased pulmonary Th1 cell frequency, only. Interferon-γ−/− mice, deficient in Th1 cells, developed a significantly enhanced fibrosis response compared to that of C57BL/6J mice. The tissue adaptive immune response influences the pulmonary disease response to radiotherapy, as an increased Th17 cell frequency enhanced and a Th1 response spared, fibrosis in mice.

## Introduction

Cancers affecting organs in the thoracic region are commonly treated with radiation, although side-effects of pulmonary fibrosis and pneumonitis, which occur in up to 15% of treated patients^1^, can limit the effectiveness of this modality. The mechanisms leading to the pathologies of excessive inflammation, characteristic of pneumonitis (also called alveolitis), or deposition of extracellular matrix in the lung interstitium (fibrosis) as a consequence of radiotherapy are incompletely understood but likely include a primary response of cell injury or death followed by a tissue response to this primary injury^2–4^. This tissue response can include the rapid production of cytokines, which, as they serve to promote the pulmonary infiltration and activation of innate and adaptive immune cells, can contribute to disease^5–7^. Evidence for a lymphocyte contribution to the tissue response to radiation injury, specifically, includes results from a study wherein mice receiving a thymectomy prior to exposure to total body irradiation showed a decreased incidence of radiation pneumonitis^8^ and reports of increased percentages of bronchoalveolar lavage lymphocytes in patients following chest radiotherapy^9–11^. The specific lymphocyte types and their involvement in the development of radiation-induced lung injuries have yet, however, to be elucidated^12^.

The pneumonitis and fibrosis responses evident clinically have been modelled in mice, which show strain and genotype dependent presentations of these traits^13–19^. Specifically, we^13, 15, 16^ and others^14, 19–21^ have documented that following thoracic irradiation inbred strains of mice vary in the times at which respiratory distress is evident, and at distress certain strains have developed pneumonitis, while others are prone to pneumonitis with fibrosis.Investigations of these lung response phenotypes have revealed post irradiation gene expression profiles associated with the pneumonitis and pneumonitis with fibrosis responding mouse strains^18, 22^. Further in mouse models, we demonstrated combined toll-like receptor 2 (*Tlr2*) and *Tlr*4 deficient mice develop an enhanced fibrotic response, of earlier onset, relative to that of wild type C57BL/6J mice, in response to thoracic irradiation^17^ and thus are a useful model for severe fibrosis. Finally, animal models of radiation-induced lung disease also present the clinically evident increased lymphocyte response^23, 24^, the extent of which may depend on mouse strain^18, 20, 21^, which facilitates the investigation of an adaptive immunity contribution to radiation-induced tissue injury.

Herein we investigated the radiation-induced responses of specific inbred and genetically altered mice to define the lymphocyte populations and thus specific pathways contributing to the development of pneumonitis or pneumonitis with fibrosis as a consequence of radiotherapy.

## Results

### Pulmonary lymphocyte populations in radiation-induced lung injury

**Figure 1:**
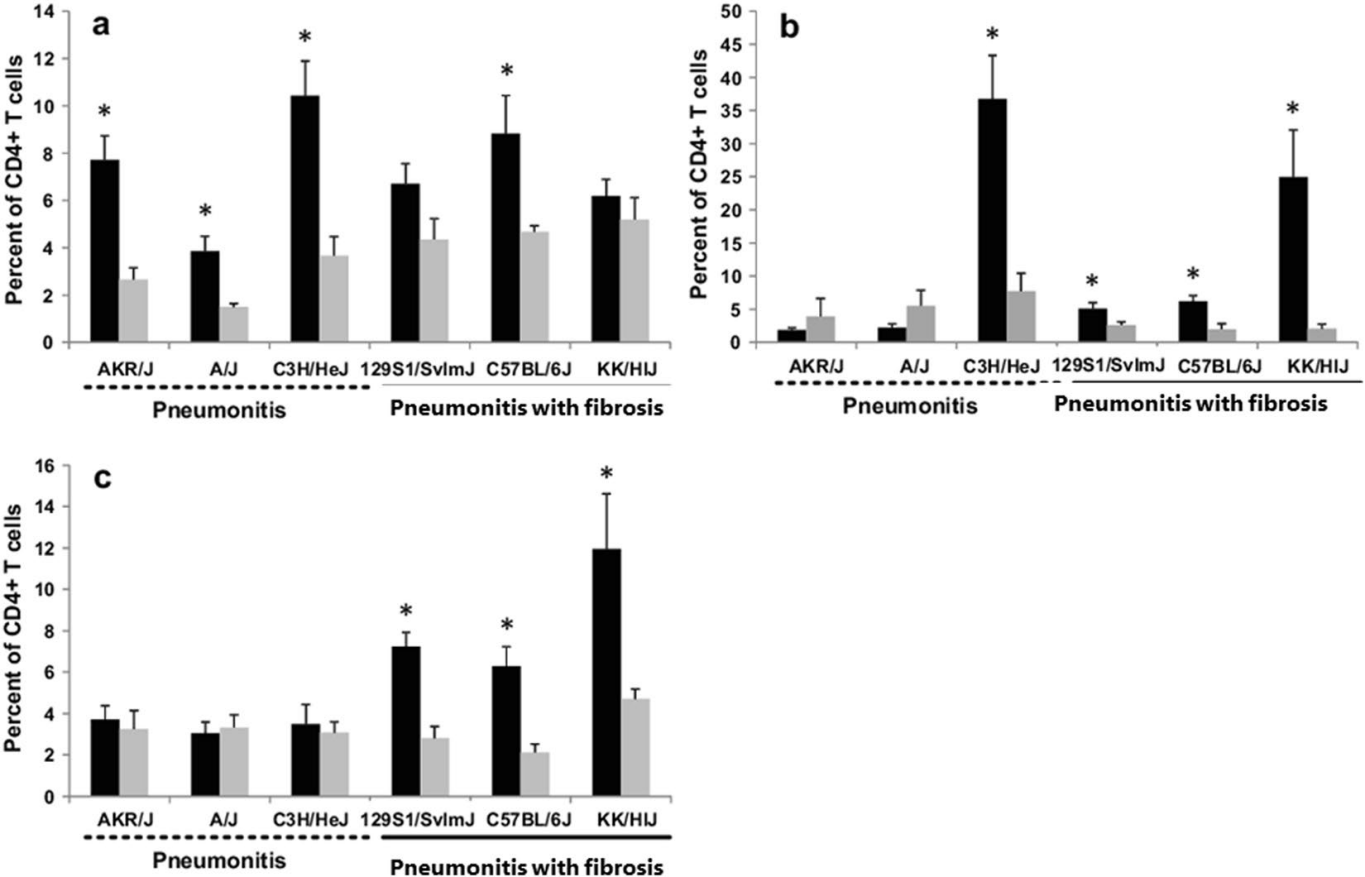
Pulmonary lymphocytes in inbred mouse strains following thoracic irradiation. Mice of 6 inbred strains received 18 Gy whole thorax irradiation and were euthanized when in respiratory distress. Shown are percentages of (**a**) Th1 (CD4+Ifnγ+), (**b**) Th2 (CD4+Il13+) and (**c**) Th17 (CD4+IL17+) cells of total lung lymphocytes in distressed (black bars) and control (grey bars) mice. Results are mean ± SE for n = 4–8 mice per group. *Indicates a significant difference compared to unirradiated control (P value < 0.05).

To investigate whether distinct lymphocyte populations could contribute to the radiation injuries of pneumonitis or pneumonitis with fibrosis, we profiled the lymphocyte populations in the lungs of 3 strains of mice we had previously determined to develop the pneumonitis phenotype (C3H/HeJ; A/J; AKR/J) and compared these data to that of three strains which we showed to develop pneumonitis with fibrosis (C57BL/6J; 129S1/SvImJ; KK/HIJ), (refs 13, 15, 16, 25–27. Mice were irradiated with 18 Gy to the thorax, euthanized upon presentation of respiratory distress and lymphocyte populations of the lung assessed with flow cytometry. The onset of distress varied from 8 weeks in KK/HIJ mice to 25 weeks in A/J mice, as shown in Supplementary Fig. S1, and was not indicative of a pneumonitis or pneumonitis with fibrosis response, in agreement with previous studies^13, 16, 27^. Of the 8 lymphocyte populations surveyed, Th17 cell frequencies, which were increased relative to control levels only in the strains prone to pneumonitis with fibrosis, best segregated the strains of known pneumonitis with fibrosis response from those previously established to develop pneumonitis alone, as shown in Fig. 1 and Supplementary Fig. S2. Th1 and Th2 cell frequencies did not completely distinguish the pneumonitis versus pneumonitis with fibrosis responses as significant increases in the frequency of Th2 cells were evident in three fibrosis prone and in one pneumonitis prone strain while Th1 cells were of greater frequency in the three strains developing pneumonitis, and in one pneumonitis with fibrosis prone line. The numbers of CD4 positive cells per lung did not differ between irradiated and control mice in each strain, with the exception of AKR/J mice where the cell number in control mice exceeded that of irradiated mice (p = 0.03, as shown in Supplementary Fig. S3). Despite this, the lungs of AKR/J mice, post irradiation, had a greater number of Th1 cells compared to control levels and treated mice did not differ in numbers of Th2 and Th17 cells, from those from control mice, consistent with the depiction in Fig. 1. Regarding the remaining cell types, a radiation-induced decrease in natural killer cells was evident for all strains, except 129S1/SvImJ mice, while no consistent differences in percentages of T, B, CD4 or CD8 cells in the lung were evident between pneumonitis prone and pneumonitis with fibrosis prone strains (Supplementary Fig. S2).

### Survival and lung disease phenotype of irradiated interleukin-17 deficient mice

**Figure 2:**
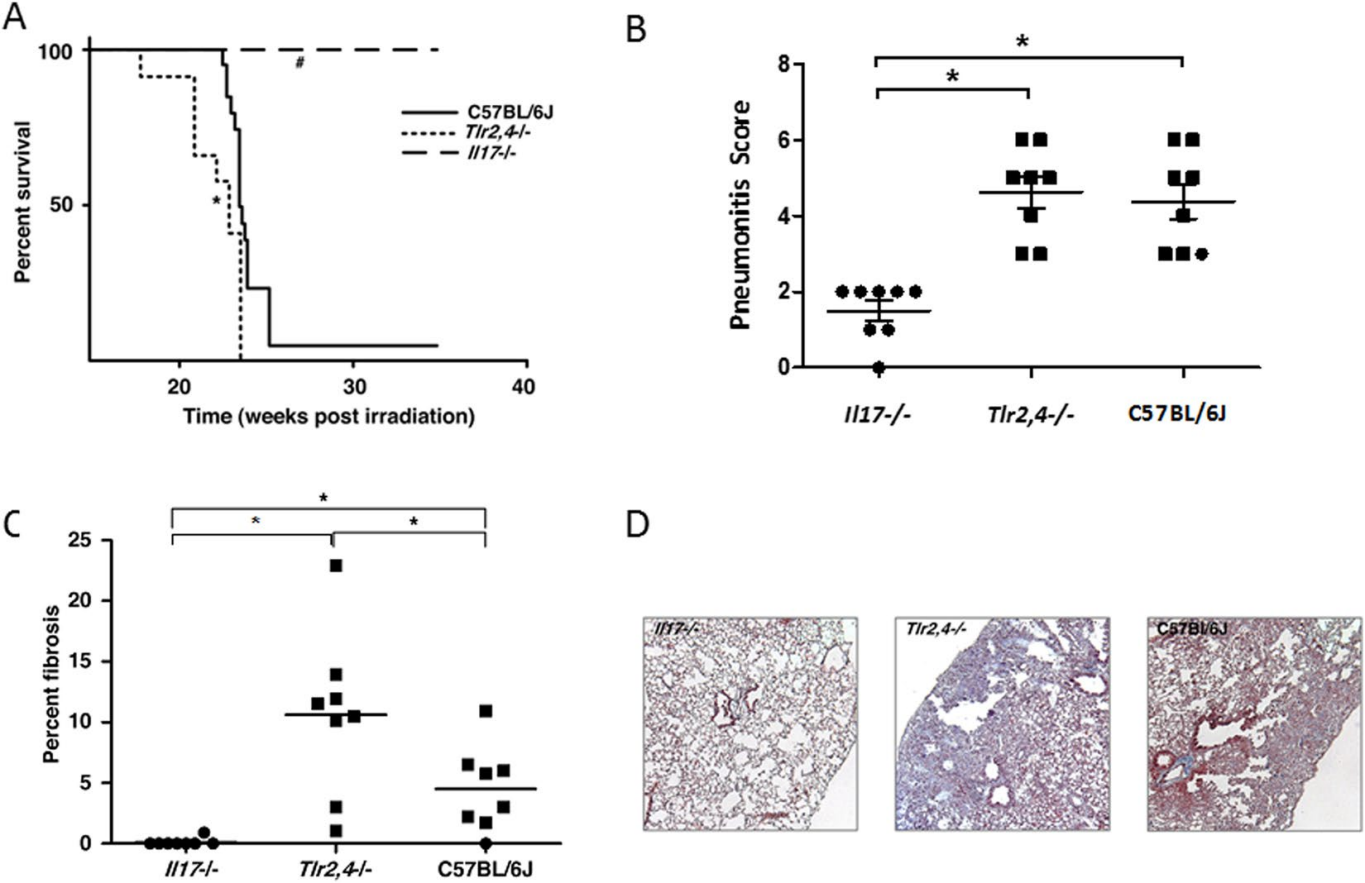
Radiation-induced pulmonary phenotype of C57BL/6J WT, *Tlr2*,*4*−/− and *Il17*−/− mice. Following a single dose of 18 Gy thoracic irradiation, mice were euthanized when in respiratory distress or at 35 weeks post irradiation which was the end of experiment (EOE). (**A**) Survival following treatment. *,^#^Indicate a significant difference in survival compared to C57BL/6J WT mice (P-value < 0.05); n = 12–20 mice per strain. (**B**) Pneumonitis scores derived from semi quantitative evaluation of histological sections. (**C**) Fibrosis scores calculated as the percent of fibrotic lung tissue in Trichrome stained histological sections. Squares indicate mice that were euthanized due to respiratory distress and circles indicate mice that survived to the EOE. *Indicates a significant difference between groups (P-value < 0.05). (**D**) Images of Masson’s trichrome-stained lung sections from strains indicating different fibrosis responses to whole thorax irradiation; magnification = 200X.

Given the increased frequency of Th17 cells in the lungs of mice manifesting distress due to radiation-induced pneumonitis with fibrosis, which was not evident in mice succumbing with pneumonitis only, we sought to determine whether a deficiency in interleukin-17A (Il17), an effector molecule of Th17 cells^28^, affects the development of radiation induced fibrotic lung disease. For this, groups of C57BL/6J, *Tlr2/*4−/− and *Il17*−/− mice were irradiated and their survival to the onset of respiratory distress, or to 35 weeks post irradiation, was recorded. As shown in Fig. 2A, mice of the inbred C57BL/6J strain developed respiratory distress at 24–26 weeks post irradiation and of the *Tlr2*,*4*−/− strain significantly earlier than C57BL/6J mice as in a prior study^17^. *Il17*−/− mice survived significantly longer than C57BL/6J mice (p < 0.0001) with all mice euthanized at the experimental endpoint of 35 weeks post irradiation, as shown in Fig. 2A. For the C57BL/6J and *Tlr2*,*4*−/− strains, all mice presenting respiratory distress were confirmed to have developed significant pneumonitis (average score = 4.3; Fig. 2B), compared to the level in untreated control mice (average score = 0.5). The average pneumonitis score of *Il17*−/− mice, euthanized at the experimental endpoint of 35 weeks post irradiation, was 1.5 ± 0.3; and significantly lower than that of C57BL/6J mice (p = 0.0002). The extent of fibrosis, evident histologically, also depended on strain with levels in irradiated *Tlr2*,*4*−/− mice exceeding those of C57BL/6J mice (Fig. 2C,D), as before^17^. No fibrosis was evident in the lungs of 7/8 *Il17*−/− mice; while one *Il17*−/− mouse developed minimal fibrosis of 0.9% of its lung tissue.

Interleukin-17 deficient mice were thus spared radiation-induced lung disease, as evidenced by the absence of a distress response and by minimal histological changes in response to a radiation dose which produced pneumonitis with fibrosis in C57BL/6J mice and earlier onset respiratory distress with enhanced fibrosis in *Tlr2/*4−/− mice.

### Radiation-induced lung disease development in Il17−/−, *Tlr2*,*4*−/−, and C57BL/6J mice

To monitor the development of the disparate lung response phenotypes groups of *Il17* and *Tlr2*,*4* deficient mice, and C57BL/6J genetic background control mice, were exposed to 18 Gy whole thorax irradiation and their lung responses assayed in mice surviving to 16, 20, 26 and 35 weeks post irradiation. As shown in Supplementary Fig. S4, minimal lung disease was evident, histologically, at the early time point of 16 weeks post irradiation in all strains. The pneumonitis and fibrosis scores increased in mice of the *Tlr2*,*4*−/− strain at 20 weeks and no mice of this strain survived to the later timepoints. In C57BL/6J mice the significant pneumonitis and fibrosis phenotype increase occurred at 20–26 weeks post irradiation in agreement with the survival data of Fig. 2. The fibrosis phenotype of *Il17*−/− mice did not differ from that of control (unirradiated) mice at any of the time points (p > 0.59), but a significant, although minimal, increase in pneumonitis was evident in these mice at 16 weeks post irradiation (p < 0.05).

**Figure 3:**
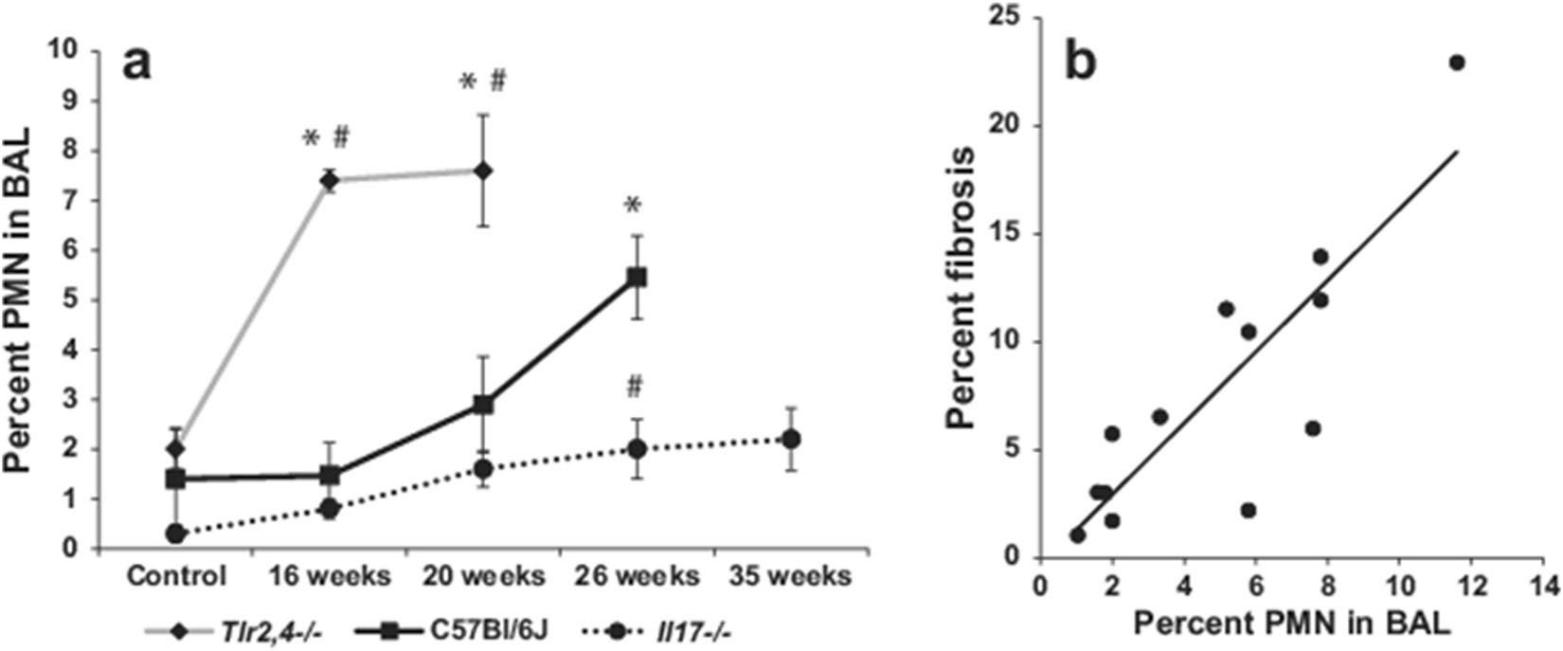
Radiation-induced pulmonary inflammatory response of C57BL/6J WT, *Tlr2*,*4*−/− and *Il17*−/− mice. Following a single dose of 18 Gy thoracic irradiation, surviving mice were euthanized 16, 20, 26 or 35 weeks later. Bronchoalveolar lavage (BAL) samples were collected at necropsy and cells were morphologically identified from cytospin preparations. (**a**) PMN percentages (mean ± SE) for groups of 4–8 mice. *Indicates a significant difference compared to corresponding control values, ^#^indicates a significant difference compared to C57BL/6J values at the same time point (P-value < 0.05). (**b**) Correlation of BAL PMN percentages and fibrosis scores in animals euthanized due to respiratory distress (p-value < 0.001).

To document the inflammatory response associated with the development (C57BL/6J and *Tlr2/*4−/− mice) or sparing (*Il17*−/− mice) of radiation-induced pneumonitis with fibrosis bronchoalveolar lavage samples from mice of the time course experiment were analyzed. Thoracic irradiation resulted in an eight to ten fold increase in the total number of cells infiltrating the lungs of the strains of mice succumbing to pneumonitis with fibrosis (C57BL/6J and *Tlr2/*4−/− mice) with the influx evident at 20 and 26 weeks post irradiation, as presented in Table 1. In *Il17*−/− animals, however, the total cell count at 16, 20, 26 and 35 weeks post irradiation was not different from control levels (p > 0.08) and was, consistently, significantly lower than that measured in distressed C57BL/6J mice (p < 0.05). The lavage of *Tlr2*,*4*−/− mice contained significantly more neutrophils than that of C57BL/6J mice at 16 and 20 weeks post irradiation (p < 0.04) while the lavage neutrophil % did not differ between these strains in mice euthanized due to distress (p = 0.26), as shown in Fig. 3a. Given the increased frequency of neutrophils detected in the lavage of mice presenting significant pulmonary fibrosis (*Tlr2*,*4*−/− mice at 20 weeks post irradiation and C57BL/6J mice at 26 weeks) we investigated the association of these traits and identified the frequency of PMNs in lavage at the time of distress to be significantly correlated with extent of fibrotic lung disease (correlation coefficient = 0.72, p value = 0.00024, Fig. 3b). In contrast, the lavage PMN percent of irradiated *Il17*−/− mice was only minimally increased compared to levels in untreated control animals at 20, 26 and 35 weeks post treatment (p < 0.05) and was significantly reduced compared to C57BL/6J levels at 26 weeks post irradiation (p < 0.04).

**Table 1:** Total bronchoalveolar lavage cell count in mice exposed to 18 Gy thoracic irradiation.

	C57Bl/6J cell count (×10^4^/ml)	*Il17*−/− cell count (×10^4^/ml)	*Tlr2*,*4*−/− cell count (×10^4^/ml)
Control	2.15 ± 0.50	4.95 ± 0.75	3.02 ± 0.69
16 weeks	2.95 ± 0.49	6.93 ± 0.44	10.59 ± 2.42^a,b^
20 weeks	40.29 ± 11.27^a^	4.18 ± 0.46^b^	24.35 ± 5.39^a^
26 weeks	34.18 ± 10.12^a^	3.17 ± 1.24^b^	
35 weeks		4.96 ± 0.63	

Data are presented as mean ± SE. of n = 4–8 mice per strain and time point. ^a^Indicates significant differences compared to control and ^b^indicates significant differences compared to C57BL/6J cell numbers at the corresponding time point.

**Figure 4:**
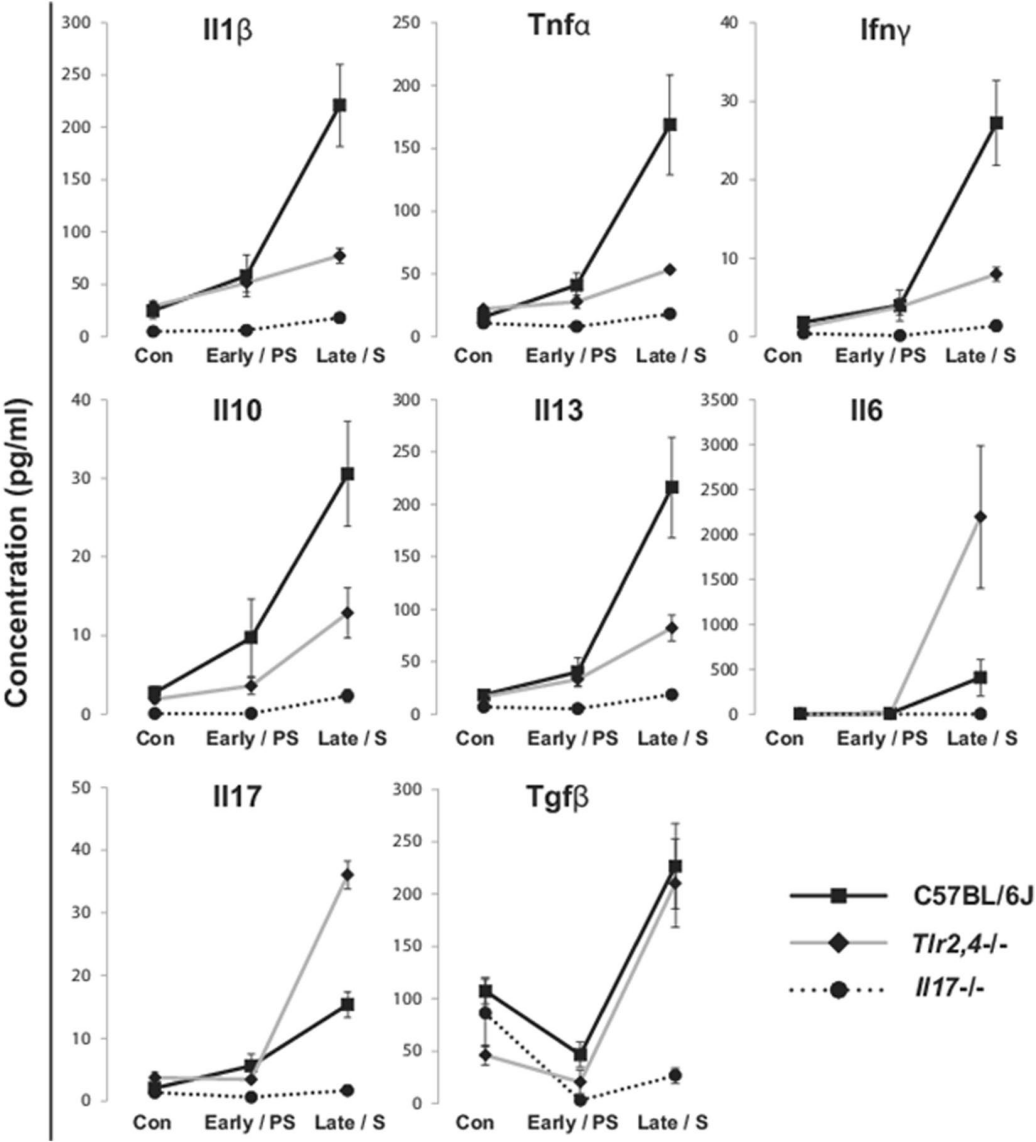
Radiation-induced bronchoalveolar lavage cytokine levels in C57BL/6J WT, *Tlr2*,*4*−/− and *Il17*−/− mice. Mice were exposed to 18 Gy thoracic irradiation and survivors euthanized at 16, 20, 26 and 35 weeks post treatment. Bronchoalveolar lavage (BAL) samples were collected at necropsy and cytokine levels measured using the Multiplex (Il1β, Ifnγ, Tnfα, Il10, Il13, Il17, Il6) and ELISA (Tgfβ) assays. Results are presented as mean ± SE for 4–8 mice. On the x axis, Early/PS (Early/Pre-symptomatic) indicates early timepoints or when the animals are not showing signs of lung damage (*Tlr2*,*4*−/−: 16 weeks; C57BL/6J WT: 16&20 weeks; *Il17*−/−: 16&20 weeks) and Late/S (Late/Symptomatic) indicates late timepoints or when the mice are in respiratory distress (*Tlr2*,*4*−/−: 20 weeks; C57BL/6J WT: 26 weeks; *Il17*−/−: 26&35 weeks).

Next, we measured the levels of 8 cytokines in the bronchoalveolar lavage of *Tlr2*,*4*−/− and C57BL/6J mice at both a presymptomatic timepoint (before distress from pneumonitis with fibrosis) and at respiratory distress and compared these data to profiling of bronchoalveolar lavage procured from *Il17*−/− mice at similar post irradiation timepoints. As shown in Fig. 4, the lavage cytokine measurements from presymptomatic mice differed only minimally from those of unirradiated mice, with a significant increase in Tnfα evident in C57BL/6J mice at this time, and of Il6, Il13 and Ifnγ in the early response of *Tlr2*,*4*−/− mice (p < 0.05). Upon presentation of distress from pneumonitis with fibrosis in *Tlr2*,*4*−/− and C57BL/6J mice, however, the lavage levels of all 8 cytokines exceeded those of unirradiated controls and showed strain dependent increases. In particular, the lavage of *Tlr2*,*4*−/− mice contained significantly greater amounts of Il6 and Il17 compared to that of C57BL/6J mice (p < 0.05), similar amounts of Tgfβ, and reduced levels of Ifnγ, Tnfα Il10, Il1β and Il13. The cytokine levels in the lavage of irradiated *Il17*−/− mice were consistently lower than those in C57BL/6J mice (p < 0.05) and were only minimally different from the levels in unirradiated controls at the later time point (26, 35 weeks post irradiation), when an increase in Il1β (p = 0.01) and a decrease in Tgfβ (p = 0.002) were evident in *Il17*−/− mice.

### Pulmonary lymphocyte populations of C57BL/6J WT, *Tlr2,4*−/− and Il17−/− mice following thoracic irradiation

**Figure 5:**
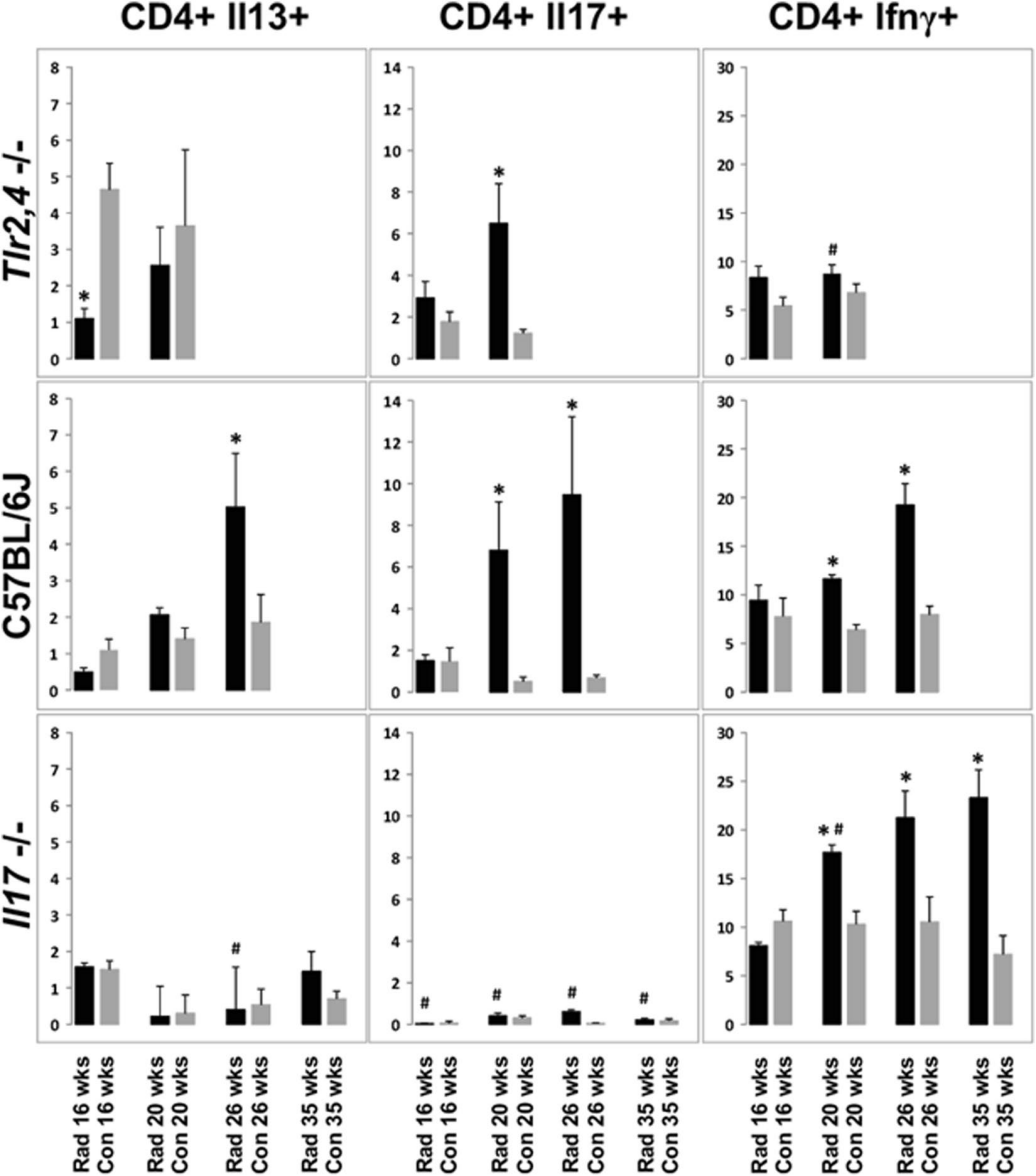
Radiation-induced pulmonary T helper cell populations in C57BL/6J WT, *Tlr2*,*4*−/− and *Il17*−/− mice. Mice were exposed to 18 Gy whole thorax irradiation and survivors euthanized 16, 20, 26 or 35 weeks later. Th1 (CD4+Ifnγ+), Th2 (CD4+Il13+) and Th17 (CD4+Il17+) cell counts were measured through flow cytometry of total lung tissue. Results are percentages of total CD4+ lymphocytes and are shown as mean ± SE for groups of 4–8 mice. *Indicates a significant difference compared to corresponding control values, ^#^indicates a significant difference compared to C57BL/6J values at the same time point (P-value < 0.05).

To investigate the lymphocytic immune response associated with susceptibility to radiation-induced pulmonary fibrosis we typed the pulmonary lymphocyte populations present during the development of the phenotype. Groups of *Il17-* and *Tlr2*,*4-*deficient mice, and C57BL/6J genetic background control mice, were exposed to 18 Gy whole thorax irradiation and lymphocyte populations were assayed in mice surviving to 16, 20, 26 and 35 weeks post irradiation. As shown in Fig. 5 and Supplementary Fig. S5, C57BL/6J mice presented with radiation-induced increases in the frequencies of Th17 (CD4+Il17+), Th2 (CD4+Il13+) and Th1 (CD4+Ifnγ+) cells during the development of the phenotype, while the most prominent change in *Tlr2*,*4* deficient mice was a significant increase in Th17 cell frequency in irradiated mice. Indeed, a significant increase in the numbers of pulmonary Th17 cells was associated with fibrosis in C57BL/6J and *Tlr2*,*4*−/− mice in that all animals succumbing to the disease displayed increased Th17 cells, peaking at the time of distress. Distressed *Tlr2*,*4*−/− mice, further, presented a significantly lower frequency of Th1 cells compared to levels in distressed C57BL/6J mice (p = 0.001) despite similar values in control animals (p > 0.16). In contrast, the lymphocyte phenotype of *Il17*−/− mice included radiation-induced increases in Th1 cells, only, which were evident at 20–35 weeks post irradiation and exceeded the response of C57BL/6J mice at the 20-week time point (p = 0.001).

A radiation-induced increase in the frequency of Il17-producing lymphocytes, apart from the CD4+ population, was also observed in *Tlr2*,*4-*deficient and C57BL/6J mice euthanized at the time of distress, compared to levels in control animals (p < 0.04), as shown in Supplementary Fig. S6. The proportion of CD4-Ifnγ+ cells among lung lymphocytes was also increased in all strains after irradiation (p < 0.03), with levels in *Il17*−/− animals exceeding the response of C57BL/6J mice.

In summary, the more severe fibrosis response of *Tlr2*,*4*−/− mice, compared to wildtype C57BL/6J mice, occurred with earlier onset neutrophilia, a reduced frequency of pulmonary Th1 cells in the presence of increased Th17 cell frequency, and greater amounts of Il6 and Il17; whereas the sparing of lung disease which occurred in the absence of Il17, featured a prominent pulmonary Th1 lymphocytic response.

### Survival and lung disease phenotypes of irradiated Ifnγ−/− mice

As we observed Il17-deficient mice to be protected from radiation-induced pneumonitis with fibrosis and to have accumulated Ifnγ-expressing Th1 cells in the post irradiation period, we sought to directly assess how a deficiency in Th1 cells would affect the radiation-induced pneumonitis with fibrosis response of C57BL/6J mice. For this, groups of *Ifnγ*−/− and C57BL/6J genetic background control mice were exposed to 18 Gy whole thorax irradiation and their lung responses and lymphocyte profiles assayed in mice upon presentation of respiratory distress.

**Figure 6:**
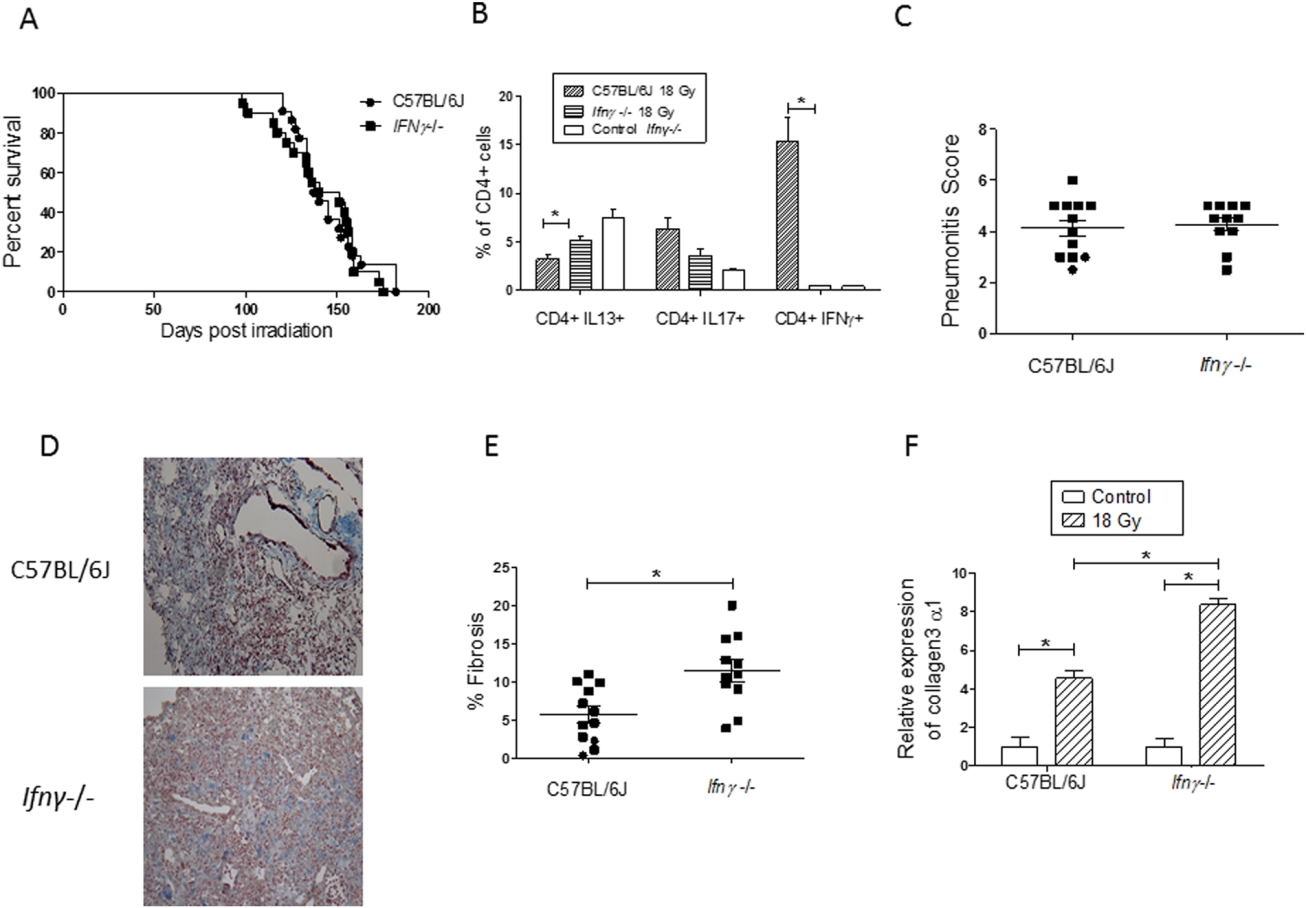
Radiation-induced pulmonary phenotype and pulmonary T helper cell populations in C57BL/6J WT and interferon-γ deficient mice. Following a single dose of 18 Gy thoracic irradiation, mice were euthanized when in respiratory distress or at 26 weeks post irradiation which was the end of experiment (EOE). (**A**) Survival following treatment did not differ by strain (P-value = 0.09); n = 20–22 mice per strain. (**B**) Th1 (CD4+Ifnγ+), Th2 (CD4+Il13+) and Th17 (CD4+Il17+) cell counts were measured through flow cytometry on total lung tissue. Results are percentages of total CD4+ lymphocytes and are shown as mean ± SE for groups of 6–9 mice. The controls are unirradiated interferon-γ deficient mice. (**C**) Pneumonitis scores derived from semi quantitative evaluation of histological sections. (**D**) Images of Masson’s trichrome-stained lung sections from strains indicating different fibrosis responses to whole thorax irradiation; magnification = 200X. (**E**) Fibrosis scores calculated as the percent of fibrotic lung tissue in Trichrome stained histological sections. Squares indicate mice that were euthanized due to respiratory distress and circles indicate mice that survived to the EOE. (**F**) The fold change in expression of collagen 3α1 in the right lungs of a subset of mice euthanized in (**A**), relative to a reference gene is given ± std. error; n = 3–6 mice per group. *Indicates a significant difference between groups (P-value < 0.05).

In response to whole thorax irradiation, the time to onset of respiratory distress of *Ifnγ*−/− mice did not differ from that of C57BL/6J mice (p = 0.09), as presented in Fig. 6A. Flow cytometric analyses of a subset of these distressed mice showed the percentages of Th17 (CD4+Il17+), Th2 (CD4+Il13+) and Th1 (CD4+Ifnγ+) cells detected in the lungs of C57BL/6J mice to agree (p > 0.25) with the data presented for this strain in Fig. 5. Also as expected, flow cytometric analyses confirmed there to be a low frequency of Th1 (CD4+Ifnγ+) cells in lung tissue of *Ifnγ*−/− mice, as illustrated in Fig. 6B. An interstrain comparison of the lymphocyte profiles identified lung tissue from *Ifnγ*−/− mice to have a significantly lower frequency of Th1 (CD4+Ifnγ+) cells (P < 0.0003), and an increased frequency of Th2 (CD4+Il13+) cells (P < 0.008; Fig. 6B) at respiratory distress, relative to levels in C57BL/6J mice, while the percentage of pulmonary Th17 (CD4+Il17+) cells did not differ by strain, (P = 0.065).

Lung disease phenotyping verified the radiation induced injury of the C57BL/6J mice to be pneumonitis with fibrosis, as shown in Fig. 6C,D,E, in agreement with data presented in Fig. 2 and prior reports^15, 17, 25, 29, 30^. Histological evaluation of the lungs of *Ifnγ*−/− mice, at distress, revealed a similar pneumonitis to that in C57BL/6J mice (p = 0.6; Fig. 6C) but that the *Ifnγ*−/− mice developed more fibrosis than levels detected in C57BL/6J mice, at distress, by both histological examination (Fig. 6D,E; p = 0.0045) and by expression of collagen 3α1 (p = 0.02), as shown in Fig. 6F. Inflammatory phenotyping of these mice showed the radiation response of the strains to be similar in terms of both total bronchoalveolar lavage cells (p = 0.98) and lavage percent PMN (p = 0.81). The extent of pulmonary fibrosis evident at respiratory distress was not correlated to lavage PMN in distressed *Ifnγ*−/− mice (correlation coefficient = −0.02).

In summary, the radiation response of Th1 cell deficient *Ifnγ*−/− mice was determined to be pneumonitis with enhanced fibrosis, an injury response occurring in the same time course as that of C57BL/6J mice, but with augmented pulmonary Th2, not Th17, cell frequency.

## Discussion

Our studies to characterize the phenotypes of pneumonitis and of pneumonitis with fibrosis, in terms of lymphocyte responses, have shown an association of Th17 cells with the presence, and of Th1 cells with the sparing, of radiation-induced pulmonary fibrosis in mice. Secondly, these studies revealed that the severe fibrosis response to radiation exposure can be induced through the activation of the Il6-Tgfβ-Il17 and Th17 pathway, with infiltration of PMN to the lung. Finally, the absence of Il17 was demonstrated to permit the survival of whole thorax irradiated mice to the experimental endpoint of 35 weeks post treatment, while a Th1 cell deficiency was shown not to affect the extent of pneumonitis or the time to onset of respiratory distress in C57BL/6J mice.

Radiation-induced pneumonitis with fibrosis was associated with both an increased frequency of Th17 cells, and with the presence of their main effector population, PMN, in the lungs of mice. Indeed, of the six inbred strains studied, pneumonitis with fibrosis-prone but not solely pneumonitis-prone mice showed an increase in pulmonary Th17 cells upon the presentation of radiation-induced lung disease. This effect was not due to a later onset of respiratory distress permitting a Th17-biased phenotype as fibrosis-prone KK/HIJ mice had the earliest onset of distress, and increased Th17 cells, while A/J mice, which develop only pneumonitis, survived to 25 weeks post irradiation with no increase in Th17 cells. Secondly, the investigation of genetically altered mice revealed Th17 cells to peak in number upon presentation of fibrosis (*Tlr2*,*4*−/− mice) and for fibrosis to be absent in *Il17*−/− mice, a model with undetectable levels of these lymphocytes. Thirdly, a limited cytokine survey revealed Il17, Il6 and Tgfβ to be the only cytokines produced by distressed *Tlr2*,*4*−/− mice in higher or equal amounts to those of C57BL/6J mice. As it has been reported that Il17 can induce Il6 production, which, through a positive feedback loop, together with Tgfβ, can promote Th17 differentiation^28, 31^, this pathway may have led to increased fibrosis in *Tlr2*,*4*−/− mice. Further, distressed mice had fibrosis scores proportional to the percentage of infiltrating PMN, a known effect of Th17 signaling^32^, which occurs in other lung disease models^33–38^. The correlation of fibrosis with lavage neutrophil level is supported by our similar findings in populations of genetically mixed mice^15, 25^, and is clinically relevant as increased lavage PMN are a known radiation pathology^11^. In addition, as we observed an increase in Il17-producing CD4- lymphocytes in *Tlr2*,*4*−/− and C57BL/6J mice there are thus other sources of this cytokine, apart from Th17 cells, in the radiation phenotype. Previous findings in related models^33, 39–42^ suggest these CD4-Il17+ cells to be γδ T cells, a population of innate immune cells capable of rapidly producing Il17 upon stimulation; although other lymphocyte sources of Il17 include NKT^43^ or CD8+ cells^44^, therefore Il17 sources, apart from Th17 cells, and their contribution to pneumonitis with fibrosis, if any, remain to be elucidated. Finally, among the immune responses of *Tlr2*,*4*−/− mice was a significant decrease in Th2 cells at 16 weeks post irradiation, a response which has been shown to augment pulmonary Th17 and neutrophil levels in an asthma model^45^ and may therefore be related to the fibrosis pathology.

The cytokine profile in distressed *Tlr2*,*4*−/− mice may indicate the contribution of an innate and adaptive response to radiation injury. Indeed, in these mice most assayed cytokines increased moderately, compared to the greater increases evident in C57BL/6J mice, possibly due to the absence of signalling through toll-like receptors 2 and 4. The exceptions were interleukins 6 & 17, which either do not require signalling through these receptors for their production, or are being produced in higher amounts in the lavage of distressed *Tlr2*,*4*−/− mice as a result of the low levels of other repressive cytokines such as Il13, *Ifnγ*, Il10. Fibrotic lung disease development is accelerated in radiation exposed *Tlr2*,*4*−/− mice and this may have occurred though enhanced interleukin-6 levels early in the disease process, possibly coupled with a decrease in Th2 cells, leading to increased Th17 and Il17 production and neutrophil infiltration at distress. Alternatively, as interleukin-6 can also influence macrophage differentiation^46^, the early increase in the cytokine in distressed *Tlr2*,*4*−/− mice could have affected the pulmonary macrophage phenotype of the mice, and the resultant fibrosis response.

The lymphocyte profiles of inbred and genetically altered mice indicated that while Th17 cells associated with the development of fibrosis, Th1 cells influenced the onset and/or severity of the fibrosis. Specifically, in addition to a prominent Th17 response, distressed *Tlr2*,*4*−/− mice had lower numbers of CD4+Ifnγ+cells, decreased Ifnγ amounts in lavage and succumbed with more severe fibrosis compared to wildtype mice. Similarly, KK/HIJ mice had the earliest onset of distress, among the strains surveyed, and displayed no increase in Th1 cells after radiation. Thus, the lack of a Th1 response in the presence of a Th17 profile equated to severe or early onset disease whereas in the wildtype strain an increase in both cell types resulted in less severe and delayed disease. The mitigating effect of Th1 cells on fibrosis was evident in the augmented fibrosis response of distressed *Ifnγ*−/− mice as well. These mice also had lower numbers of pulmonary CD4+Ifnγ+cells compared to wildtype mice, but in this model the more severe fibrosis may have been due to the increased frequency of pulmonary Th2 cells, a known attribute of this strain^47^, which may influence susceptibility to pulmonary fibrosis^48, 49^. The injury response of C57BL/6J mice post radiation was in this way similar to that which develops after a bleomycin challenge, which also includes pulmonary fibrosis and increases in Th1,2&17 cells^34, 35, 50^ wherein Th2&17 lymphocytes have been associated with increased disease severity and Th1 cells with a reduction in the fibrosis phenotype^50, 51^. A direct interaction of lymphocyte populations may be occurring as O’Connor *et al*.^52^ reported that *in vitro* Il17 treatment of naïve CD4+ T cells cultured in Th1 polarizing conditions has a suppressive effect on Tbet expression and Th1 development.

The Th1 response in C57BL/6J mice dampened the fibrotic response to radiation, but appears dispensable to the pneumonitis response in this strain. Indeed, distressed *Ifnγ*−/− mice, which again had lower numbers of pulmonary CD4+Ifnγ+cells compared to wildtype mice, did not differ from wildtype mice in histologically evident pneumonitis, or in related traits of time to onset of distress from whole thorax irradiation and total bronchoalveolar lavage cell counts. Thus a Th1 response is not necessary for pneumonitis development in C57BL/6J mice. Further, the prominent Th1 profile of *Il17*−/− mice, which were spared an overt lung injury, indicates a Th1 response is not sufficient for pneumonitis development in C57BL/6J mice. Whether the increased Th1 response, which was evident in all three inbred strains prone to pneumonitis alone (AKR/J, C3H/HeJ, A/J), is instrumental to the development of pneumonitis in these strains, in addition to sparing fibrosis, is not known.

Finally, in response to a dose of whole thorax irradiation to which only 5% of C57BL/6J wildtype mice survived beyond 6 months, 100% of *Il17*−/− mice were alive more than 8 months later and had developed only a minimal lung injury at this time. Interleukin-17 is therefore implicated in the pathology of pneumonitis as well as in fibrosis, a finding which is supported by one report wherein anti Il17a monoclonal antibody treatment was shown to prolong the survival of whole thorax irradiated C57BL/6J mice^53^. More comprehensive evaluation of the mechanism(s) through which Il17 may influence the onset as well as type of radiation injury developing in the lung is needed, but an effect on the Th1/Th17 lymphocyte balance, as was argued for fibrosis, and has been shown in graft versus host disease^54^, may be involved. For example, the reduced lung inflammation and fibrosis, and prolonged survival of whole thorax irradiated B6.*Ccr1*−/− mice were associated with increased *Ifnγ* gene expression^55^, which is suggestive of the Th1 phenotype of *Il17*−/− mice. In addition, a Th1/Th17 lymphocyte balance wherein Th1 cells mitigate the onset of distress only in an enhanced Th17 environment could have resulted in the survival of irradiated *Ifnγ*−/− mice wherein the lack of a sparing Th1 response in these mice (due to the Ifnγ deficiency) did not translate into an earlier onset of distress, as Th17 cell frequency was not increased in these mice.

Following the same stimulus (i.e. thoracic radiation), different mouse strains, representing the clinical spectrum of radiation injury, responded with distinct Th1/2/17 lymphocyte profiles, which ultimately dictated the presence or absence, and extent, of fibrosis. Specifically, we found the presence of Th17 cells to predict for fibrosis development whereas Th1 cells had a protective effect. Th2 cells were also associated with fibrosis development, in the absence of a Th1 response. As we, further, identified the levels of Il17 and Il6 in the bronchoalveolar lavage, a clinically accessible tissue, to be associated with severe lung disease this pathway may have potential for both diagnostic and therapeutic intervention.

## Methods

### Mice

Mice of inbred strains A/J, AKR/J, C3H/HeJ, C57BL/6J, 129S1/SvImJ, and KK/HIJ, and B6.129S7-Ifngtm1Ts/J mice were purchased from the Jackson Laboratory (Bar Harbor, USA) and housed in the Meakins-Christie Laboratories. Mice of other strains, on the C57BL/6J background, were obtained through collaboration (*Tlr2*,*4*−/− mice from Dr. Qureshi of McGill University) or a material transfer agreement (interleukin-17 (*Il17*)−/− mice from Dr. Iwakura of the University of Tokyo, ref. 56) and experimental mice bred in house. All mice were handled according to guidelines and regulations of the Canadian Council on Animal Care. Specifically, the animal experiments were performed in accordance with protocol 5601 approved by the Animal Care Committee of the Research Institute of the McGill University Health Centre.

### Thoracic irradiation and experimental groups

At the age of 8–10 weeks, female mice were exposed to a single dose of 18 Gy to the thorax using a Gamma Cell Cesium-137 source as previously described^13, 15, 17, 25, 26^. Inbred strain mice were weighed weekly from 6 weeks after irradiation, euthanized when they showed signs of distress (ruffled fur, accelerated breathing, hunched posture, weight loss > 15% of body weight), and assayed with pulmonary flow cytometry. Groups of C57BL/6J, *Tlr2*,*4*−/− and *Il17*−/− mice were, similarly, euthanized when they showed signs of distress or at specific timepoints (16, 20, 26 and 35 weeks) post irradiation. These mice were randomly divided into 2 experiments: one group of mice was assayed by pulmonary flow cytometry and the second group by bronchoalveolar lavage and histologically. Control mice were not irradiated and were euthanized at matching timepoints.

In one experiment, 8–10 week old female B6.129S7-Ifngtm1Ts/J (*Ifnγ*−/−) and C57BL/6J mice were exposed to a single dose of 18 Gy to the thorax using a Faxitron instrument as previously described^57–60^, and euthanized when they showed signs of distress. The mice were randomly divided into 2 experiments: one group of mice was assayed by pulmonary flow cytometry and the second group by bronchoalveolar lavage, pulmonary gene expression and histologically. Control mice were not irradiated and were euthanized at the 22 week timepoint.

### Lung histopathology and bronchoalveolar lavage (BAL) fluid analysis

Bronchoalveolar lavage collection was performed by cannulating the trachea and instilling the lungs with 1 mL phosphate buffered saline. The lungs were then removed and the single left lobe was perfused with 10% buffered formalin and processed histologically. 5 µm lung sections were stained with Masson’s Trichrome and the fibrosis score was calculated as the lung surface covered by fibrosis relative to the total lung surface using the Image Pro Plus software^13, 17^. To determine the degree of pneumonitis, lung sections were stained with hematoxylin and eosin and evaluated, semi-quantitatively, through blinded subjective scoring. A score of 0–6 was given, 0 being no pneumonitis and 6 being severe pneumonitis, as in previous studies^15, 25^.

The BAL fluid was centrifuged (300 g for 10 minutes at 4 °C) and the supernatant was stored at −80 °C. The cellular pellet was re-suspended in 125 µL phosphate buffered saline. Inflammatory cell counts were performed at 400X magnification on centrifuged cells (214.2 g for 3 minutes) following staining with hematoxylin-eosin (Hema-3 Stain Set) and are reported as percentage of 500 counted cells.

BAL supernatants were profiled using a custom 8-plex Bioplex Pro Cytokine kit (BioRad) containing the following cytokines: Ifnγ, Tnfα, Il1β, Il4, Il10, Il6, Il17, Il13. Tgfβ levels were measured using a Tgfβ platinum ELISA kit (eBioscience) after performing a 1:1.5 dilution of the sample with the assay buffer provided by the manufacturer.

### Lymphocyte profiling

At necropsy both lungs were cut into small pieces and placed in phosphate buffered saline containing 1 mg/mL collagenase (Sigma-Aldrich) and 1 mg/mL DNase (Roche) at 37 °C for 45 minutes. The tissue was further disrupted using a Cell Dissociation Kit (Sigma-Aldrich) and the total number of cells retrieved was determined using a Hemacytometer. Cells were stained using the following antibodies (eBioscience): CD3 APC or PE, CD19 APC, CD4 PE, CD8 PE, CD49b FITC. The following populations were measured: T cells (CD3+), B cells (CD19+), Th cells (CD3+CD4+), cytotoxic T cells (CD3+CD8+), natural killer cells (CD3−CD49b+). The populations presented in Fig. 1 were stained using CD4 APC and either Ifnγ, Il13 or Il17 PE. The populations in Figs 5 and 6 were stained using CD4 APC, Ifnγ PE-Cy7, Il13 FITC and Il17 PE.

For detection of intracellular cytokines, the cells were incubated at 1 × 10^6^ cells/mL in complete media (37 °C, 4 hours) in the presence of 50ng/mL Phorbol myristate acetate (Sigma-Aldrich), 1 µg/mL ionomycin (Sigma-Aldrich) and 1 µl/mL Golgi Stop (BD Biosciences). After activation, the cells were washed and stained with the corresponding surface antibodies and then fixed and permeabilized using Cytofix/Cytoperm (BD Biosciences). Subsequently intracellular staining was performed and the cells were acquired using FACSCalibur or LSR II cytometers. The lymphocyte population was identified based on size and granularity on a forward scatter/side scatter plot and the analysis of cell counts was completed using the FlowJo software (TreeStar, Inc).

### Quantitative real-time PCR

Following sacrifice, right lungs were immediately homogenized in 2 mL of Trizol reagent and placed in dry ice. The homogenates were stored at −85 °C until RNA isolation was completed following the manufacturer’s (Sigma, Oakville, Ontario, Canada) instructions. For gene expression analysis, 4–5 µg of total RNA from the right mouse lung was reversely transcribed with oligo(dT)12–18Primer using Superscript™ II RNase H– Reverse Transcriptase (Invitrogen, Carlsbad, CA, USA) to make cDNA. Quantitative real-time polymerase chain reaction (PCR) assays were performed using the Applied Biosystems International Prism 7500 Sequence Detection System (Foster City, CA USA) and TaqMan gene expression assays for collagen 3α1 Mm01254476_m1 and for the reference gene Atxn10 assay Mn00450332_m1 were used.

### Data analysis

Differences in survival were assessed with log-rank tests using GraphPad Prism software (www.graphpad.com). Phenotypic differences among groups were evaluated using 2-sided T tests which were performed using Microsoft Excel software. Correlation tests were performed using the cor.test function in R (http://cran.r-project.org). Gene expression data were analysed as in refs 59, 61.

### Data availability

All data generated or analysed during this study are included in this published article (and its Supplementary Information files).

## Electronic supplementary material

Supplementary Information
