# Supplementary material for: The Th1/Th17 balance dictates the fibrosis response in murine radiation-induced lung disease
Source: Sci Rep. 2017 Sep 14;7:11586. doi: 10.1038/s41598-017-11656-5 (PMC5599556; doi:10.1038/s41598-017-11656-5)
Supplement: Supplementary file 1 — Supplementary Information [file 41598_2017_11656_MOESM1_ESM.pdf]

The Th1/Th17 balance dictates the fibrosis response in murine radiation-induced lung disease

Alexandra Paun[‡] Marie-Eve Bergeron[†] and Christina K. Haston[*]

Meakins-Christie Laboratories and the Departments of [‡]Human Genetics and [†]Medicine, McGill University, Montreal, PQ, Canada

[*]Physics Department, I.K. Barber School of Arts and Sciences The University of British Columbia Okanagan, Kelowna, BC Canada

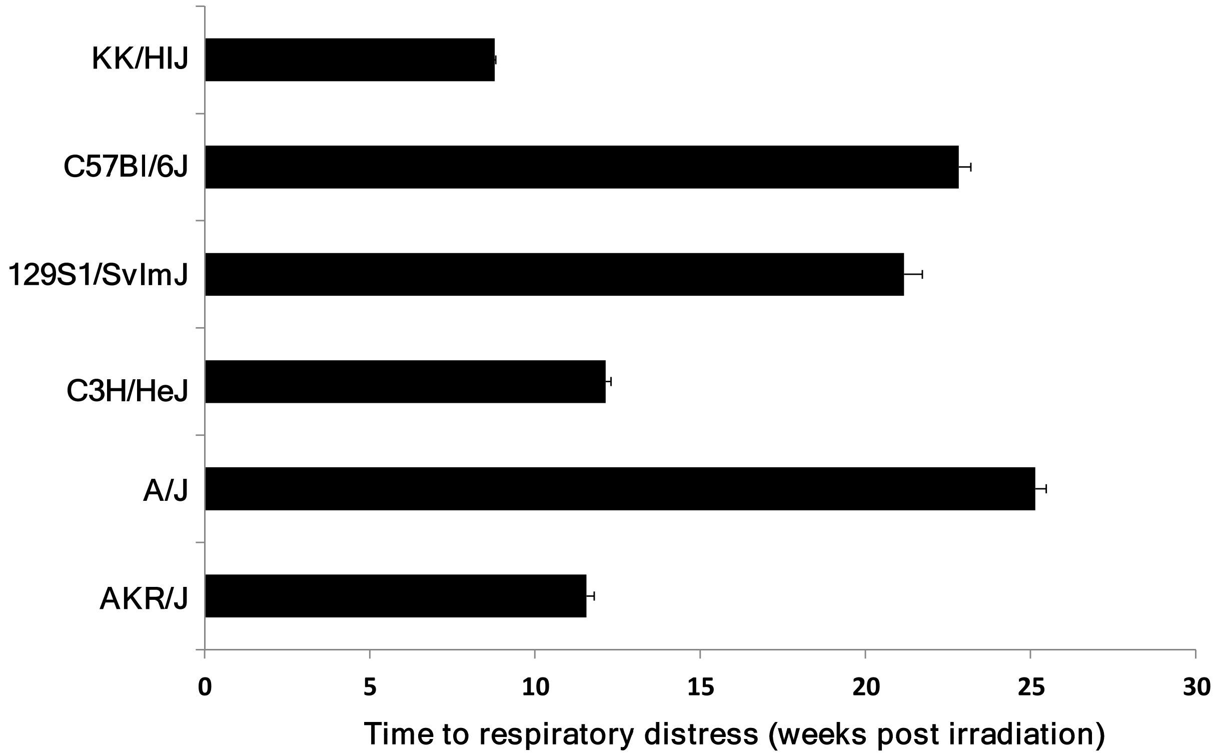

Supplementary Figure S1: Onset of respiratory distress in inbred mice following whole thorax irradiation.

Mice of 6 inbred strains received 18 Gy whole thorax irradiation and were euthanized when in respiratory distress. Survival times are shown for each strain as mean±SE for groups of 4-8 mice. KK/HIJ, C57BL/6J and 129S1/SvImJ mice are prone to pneumonitis with fibrosis, while C3H/HeJ, A/J and AKR/J mice are prone to pneumonitis.

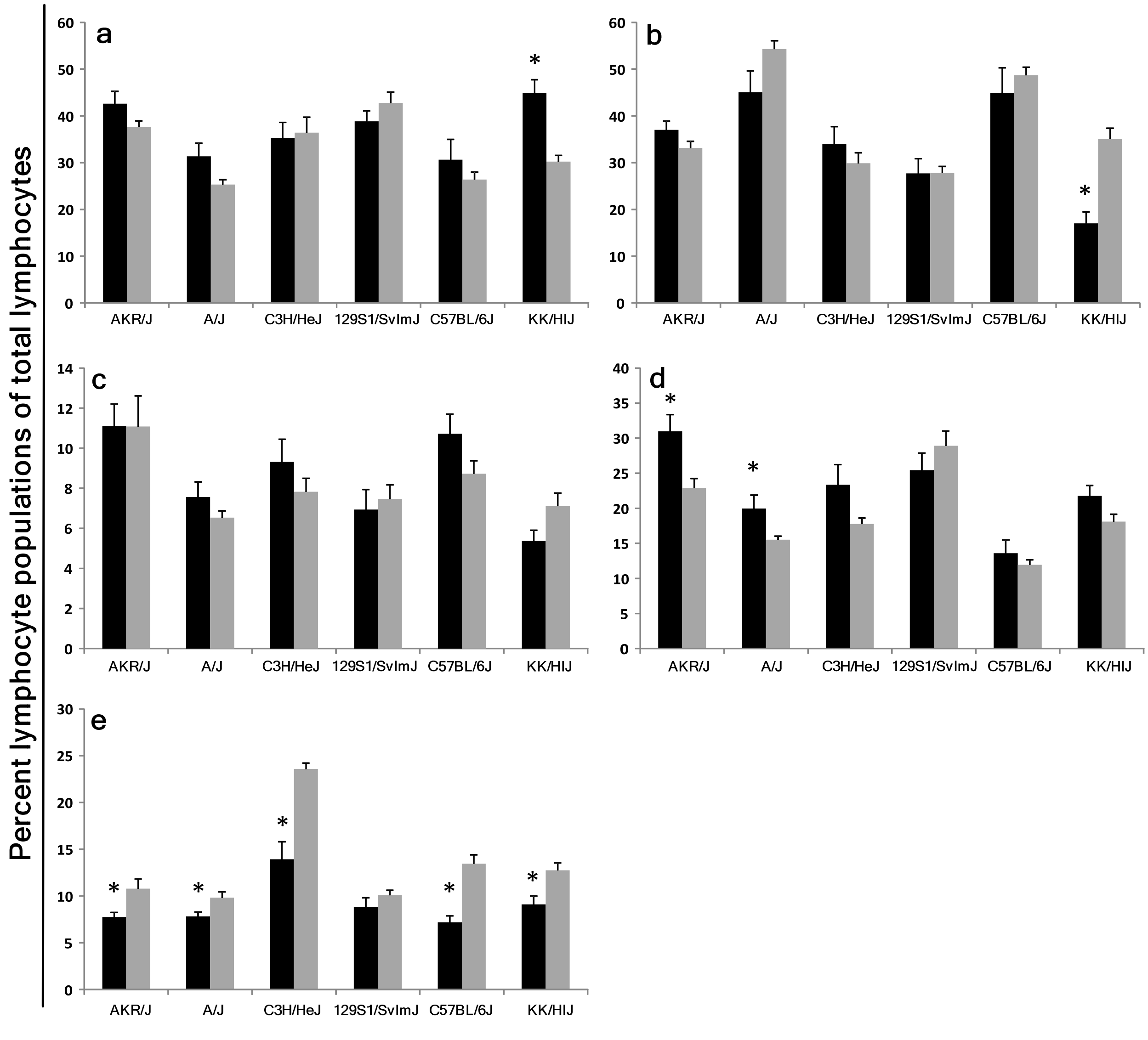

Supplementary Figure S2: Lymphocyte populations in the lungs of inbred mice following whole thorax irradiation. Mice of 6 inbred strains received 18 Gy whole thorax irradiation and were euthanized when in respiratory distress. Through flow cytometry different lymphocyte populations were analyzed in the lung tissue from treated mice (black bars) and unirradiated controls (grey bars): a) CD3+; b) CD19+ c) CD3+CD8+; d) CD3+CD4+; e) CD49b+CD3- cells. The results are percentages of total lymphocytes and are shown as mean±SE for groups of 4-8 mice. * denotes a significant difference compared to unirradiated control (Pvalue<0.05).

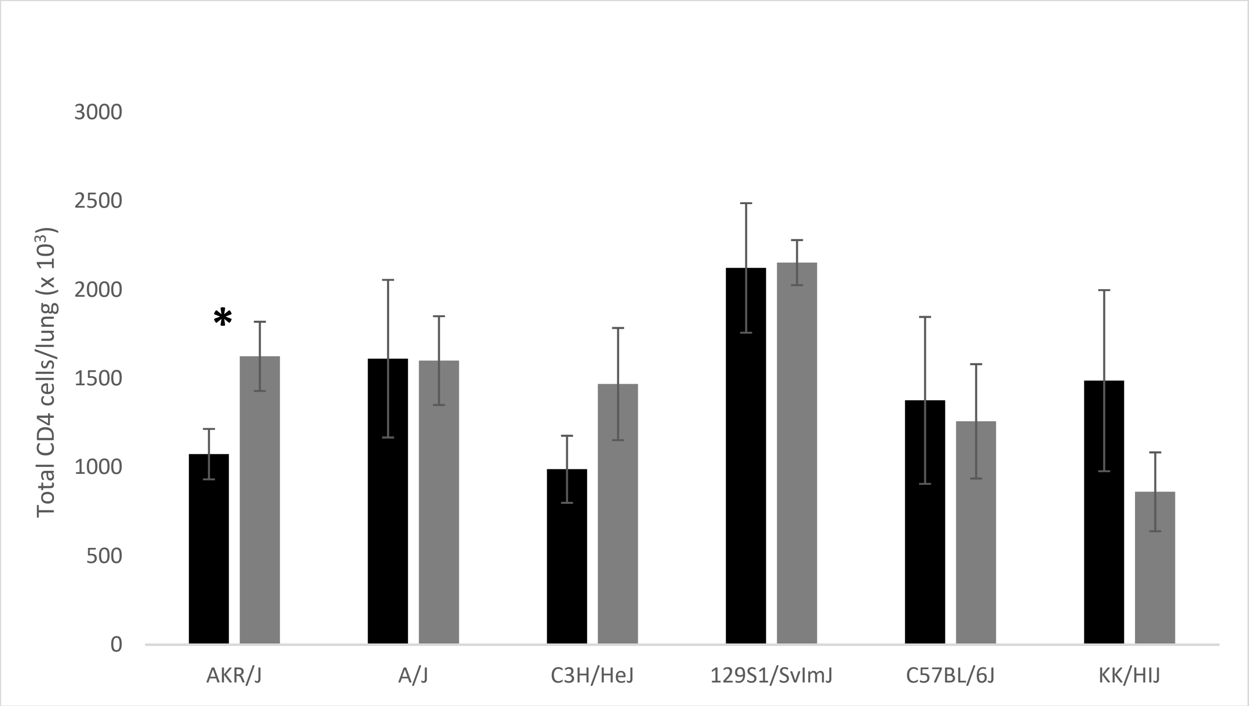

Supplementary Figure S3: Pulmonary CD4 lymphocytes in inbred mouse strains following thoracic irradiation.

Mice of 6 inbred strains received 18 Gy of whole thorax irradiation and were euthanized when in respiratory distress. Shown are numbers of CD4+ cells in distressed (black bars) and control (grey bars) mice. Results are mean±SE for n=4-8 mice per group. * indicates a significant difference compared to unirradiated control (T test pvalue<0.05).

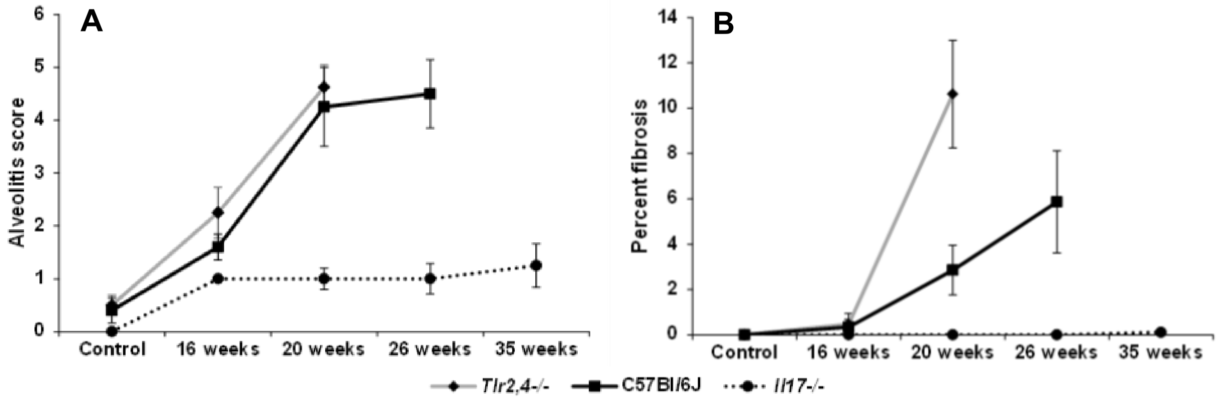

Supplementary Figure S4: Radiation-induced lung phenotype development in C57BL/6J WT, Tlr2,4-/- and Il17-/- mice. Following a single dose of 18 Gy thoracic irradiation, surviving mice were euthanized at 16, 20, 26 or 35 weeks post irradiation. A) Pneumonitis scores derived from semi quantitative evaluation of histological sections. B) Percent of fibrotic lung tissue in Trichrome stained histological sections. Control values are from unirradiated mice. Phenotypes are presented as mean±SE for groups of 5-10 mice.

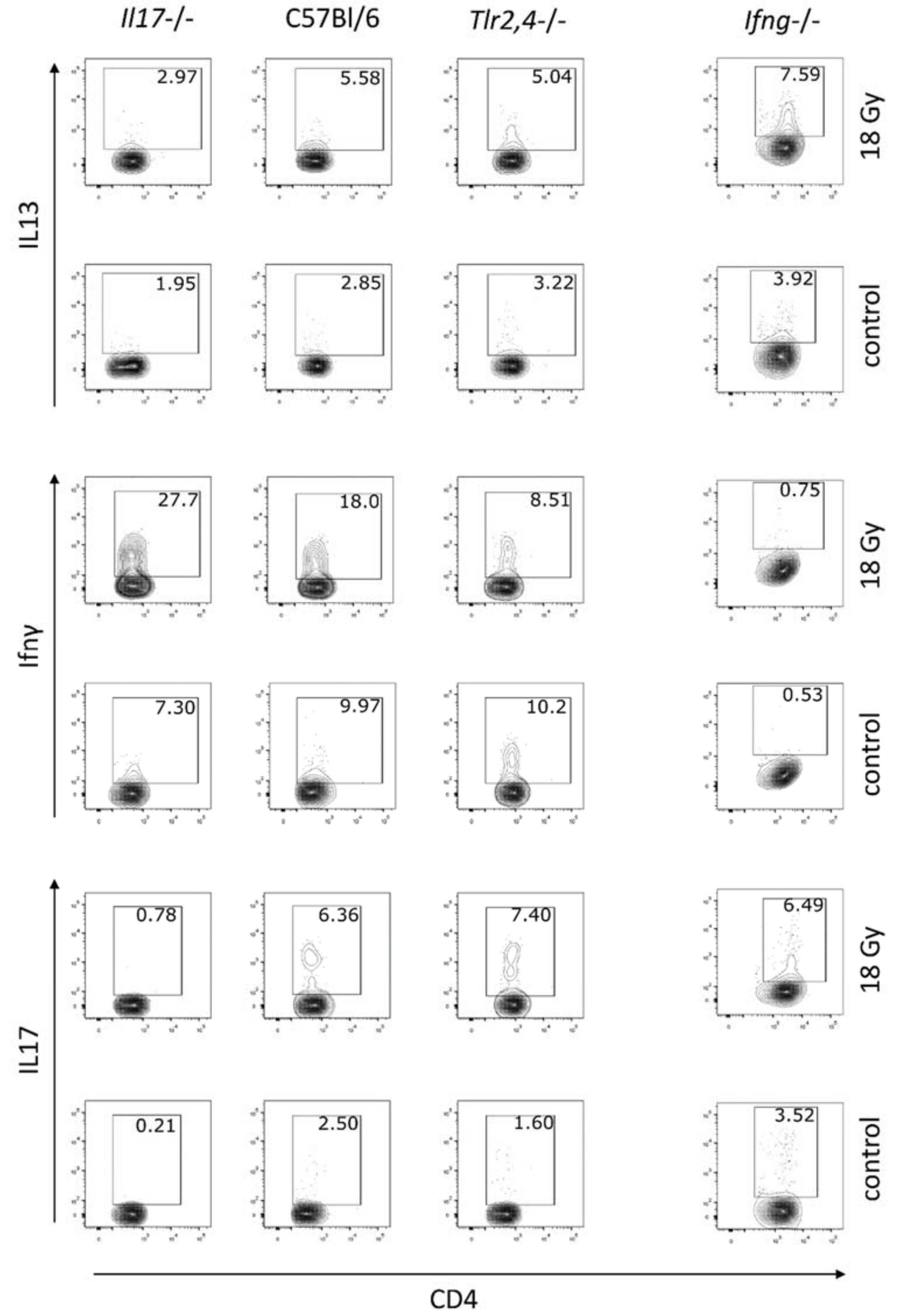

Supplementary Figure S5: Lymphocyte populations in the lungs of mice following whole thorax irradiation. Representative scatter plots of pulmonary tissue from genetically altered mice. Mice received 18 Gy whole thorax irradiation and were euthanized when in respiratory distress. Through flow cytometry different pulmonary T helper cell subsets from treated mice and unirradiated controls were analyzed. Shown are representative scatter plots of gated CD4+ lymphocytes, in control and treated mice at the latest time point after irradiation (20 weeks for Tlr2,4-/-, 26 weeks for C57BL/6J and Ifnγ-/- mice, and 35 weeks for Il17-/- mice). The numbers in the top right corner represent percentages of Th17, Th1 and Th2 cells of total CD4+ cells.

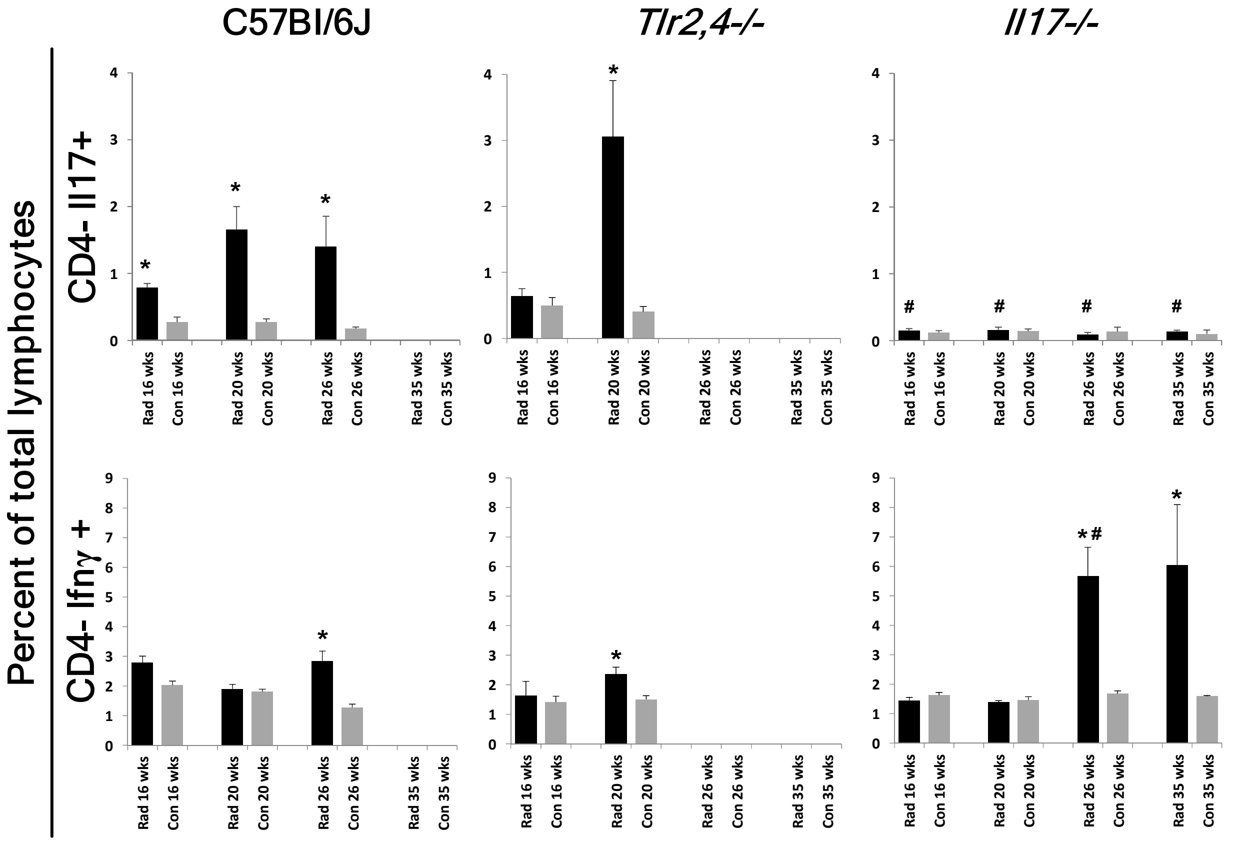

Supplementary Figure S6: CD4-Il17+ and CD4-Ifnγ+ lymphocytes in C57BL/6J WT, Tlr2,4-/- and Il17-/- mice following whole thorax irradiation. Following a single dose of 18 Gy thoracic irradiation, surviving mice were euthanized at 16, 20, 26 or 35 weeks post irradiation. CD4-Il17+ and CD4-Ifnγ+ cells were counted through flow cytometry on total lung tissue of irradiated (black bars) and control (grey bars) mice. Results are percentages among total lymphocytes shown as mean±SE for groups of 4-8 mice. * indicates a significant difference compared to corresponding control values, # indicates a significant difference compared to C57BL/6J values at the same time point (P value<0.05).
